# Does the Gut Microbiome Play a Role in Obesity in Type 1 Diabetes? Unanswered Questions and Review of the Literature

**DOI:** 10.3389/fcimb.2022.892291

**Published:** 2022-07-08

**Authors:** Heba M. Ismail, Carmella Evans-Molina

**Affiliations:** ^1^ Department of Pediatrics and the Herman B Wells Center for Pediatric Research, Indiana University School of Medicine, Indianapolis, IN, United States; ^2^ Center for Diabetes and Metabolic Diseases, Indiana University School of Medicine, Indianapolis, IN, United States; ^3^ Department of Medicine, Indiana University School of Medicine, Indianapolis, IN, United States; ^4^ Department of Biochemistry and Molecular Biology, Indiana University School of Medicine, Indianapolis, IN, United States; ^5^ Richard L. Roudebush Veterans Affairs (VA) Medical Center, Indiana University School of Informatics and Computing, Indianapolis, IN, United States

**Keywords:** type 1 diabetes, microbiome, obesity, gut dysbiosis, prebiotics, probiotics

## Abstract

Evidence suggests that type 1 diabetes (T1D) risk and progression are associated with gut bacterial imbalances. Children with either T1D or islet antibody positivity exhibit gut dysbiosis (microbial imbalance) characterized by lower gram-positive to gram-negative gut bacterial ratios compared to healthy individuals, leading to a pro-inflammatory milieu. In addition, specific gut microbiome changes, including increased virulence factors, elevated phage, prophage, and motility genes, and higher amplitude stress responses, have been identified in individuals who have or are progressing towards T1D. Additionally, gut microbiome differences are associated with and thought to contribute to obesity, a comorbidity that is increasingly prevalent among persons with T1D. Obesity in T1D is problematic because individuals with obesity progress faster to T1D, have reduced insulin sensitivity compared to their lean counterparts, and have higher risk of complications. Animal and human studies suggest higher relative abundance of bacterial taxa associated with changes in bile acid and short chain fatty acid biosynthesis in obesity. However, it is unknown to what extent the gut microbiome plays a role in obesity in T1D and these worse outcomes. In this review, we aim to evaluate potential gut microbiome changes and associations in individuals with T1D who are obese, highlighting the specific gut microbiome changes associated with obesity and with T1D development. We will identify commonalities and differences in microbiome changes and examine potential microbiota-host interactions and the metabolic pathways involved. Finally, we will explore interventions that may be of benefit to this population, in order to modify disease and improve outcomes.

## Introduction

Type 1 diabetes is associated with long-term complications including cardiovascular disease and is the leading cause of kidney failure and retinopathy (Varma et al., 2016; Centers for Disease Control and Prevention, 2019). Obesity in type 1 diabetes (T1D) is problematic because it further increases the risk for complications and mortality (Conway et al., 2009). There is increasing evidence suggesting that type 1 diabetes (T1D) risk and progression are associated with gut microbial imbalance (Brugman et al., 2006; de Kort et al., 2011; Brown et al., 2011; Giongo et al., 2011; de Goffau et al., 2013; Murri et al., 2013; de Goffau et al., 2014; Mejía-León et al., 2014; Kostic et al., 2015; O'Callaghan and van Sinderen, 2016; Zheng et al., 2018). Separately, gut bacterial imbalances are associated also with obesity (Musso et al., 2010). The development of both obesity and T1D are complex processes that include the interaction between genetic susceptibility, immune system activation or inflammation, and environmental factors, all of which are poorly understood. The gut microbiome is becoming increasingly recognized in each condition as an important link connecting genes to the environment and immune system activation.

Obesity is now prevalent (up to 46.1% in some populations) and increasing among children and adults with T1D (Nathan et al., 2009; Conway et al., 2010; Liu et al., 2010; Redondo et al., 2016; Minges et al., 2017; Corbin et al., 2018) (**Table 1**). This trend is especially problematic, as obesity is associated with worse T1D outcomes, including higher risk for short term complications, such as hypoglycemia and long-term complications, such as cardiovascular disease (Redondo et al., 2012; Redondo et al., 2016; Ferrara et al., 2017; Corbin et al., 2018). However, it is unknown to what extent the gut microbiome plays a role in obesity in T1D and associated adverse outcomes. Whether and how the gut microbiome influences obesity trends in T1D similar to what has been shown in the general population is an important area to investigate. In this review, we will explore the gut microbial compositions associated with T1D and obesity and discuss how the gut microbiome may be contributing to the rising rate of obesity in T1D by reviewing the current literature. We will then summarize the literature focused on potential therapeutic effects of intestinal microbiome manipulation.

**Table 1:** Table summarizing data on prevalence of overweight and obesity in T1D over the years.

References	Cohort/Study design	Key findings
Nathan et al, 2009 (Nathan et al., 2009)	Analysis of 1441 of the Diabetes Control and Complications Trial (DCCT) and its long-term observational follow-up, the Epidemiology of Diabetes Interventions and Complications (EDIC) study	Intensive therapy showed an association with increasing prevalence of obesity (body mass index ≥30), from 1% of subjects at the DCCT baseline (secondary to eligibility criteria) to 31% at EDIC year 12.
Conway et al, 2010 (Conway et al., 2010)	Assessed temporal patterns in overweight and obesity and predictors of weight change in 589 individuals from the Pittsburgh Epidemiology of Diabetes Complications (EDC) Study, a cohort of childhood onset T1D.	Overall 3.4% of the participants 18 years and older were obese at baseline. By 2004–2007 this had risen seven-fold to 22.7%. The prevalence of being overweight, but not obese, rose from 28.6% in 1986– 1988 to 42.0% in 2004–2007, a 47% increase.
Liu et al, 2010 (Liu et al., 2010)	Participants with diabetes (n=3953) were examined in 2001-2004 for the SEARCH for Diabetes in Youth study (SEARCH) and nondiabetic participants (n=7666) were examined during the same years of the National Health and Nutrition Examination Survey (NHANES), aged 3-19 yr	that 22.1% of youth with T1D were overweight compared with 16.1% of youth without T1D from the NHANES.
Redondo et al, 2015 (Redondo et al., 2016)	Studied 11,348 children 2 to <18 years of age enrolled in T1D Exchange between September 2010 and August 2012 with type 1 diabetes for ≥1 year and BMI ≥ 5th age-/sex-adjusted percentile.	Of the 11,348 participants, 22% were overweight and 14% obese.
DuBose, 2015 (DuBose et al., 2015)	Participants (age 2-<18 years and ≥1 year duration of T1D) enrolled in the T1D Exchange (T1DX, n=11,435) and the Diabetes Prospective Follow-up (DPV, n=21,501).	Participants in both registries (particularly from Germany, Austria, and the United States) had median BMI values that were greater than international and their respective national reference values. BMI z-scores were significantly higher in the T1DX versus the DPV (p<0.001).
Minges et al, 2017 (Minges et al., 2017)	Analyzed baseline data obtained from 5529 adolescents with T1D (mean age=15.4 ± 1.4years, 51.8% male, 77.9% white, mean HbA1c=8.7 ± 1.8%; 72mmol/mol) from the T1D Exchange Clinic Registry.	Overweight (22.9%) and obesity (13.1%) were prevalent in the overall sample and was highest among girls (40.8%) and adolescents of Hispanic/Latino race/ethnicity (46.1%). Higher prevalence seen in black/African Americans descent (17.9%) and Hispanic (15.9%) between 2010 and 2012.

## The Gut Microbiome and Metabolic Disease

### Obesity and the Gut Microbiome

The rates of obesity worldwide have tripled since 1975 (WHO, 2018). Over 1.9 billion adults are overweight and at least 650 million of those are obese. Similarly, about 39 million children under the age of 5 years and 340 million children and adolescents between 5-19 years of age globally are either overweight or obese (WHO, 2018). Gut microbiome differences and dysbioses have been associated with overweight, obesity and insulin resistance in non-diabetic children and adults (Cani et al., 2007; Turnbaugh et al., 2009; Musso et al., 2010; Del Chierico et al., 2018; Peters et al., 2018).

It is possible that obesity induces changes in the gut microbiome since earlier studies have shown that increased body fat or obesity is associated with a significantly elevated *Firmicutes : Bacteroidetes* (F:B) ratio compared to lean individuals (Dreyer and Liebl, 2018), even in the presence of similar patterns of food and energy consumption between the groups (Ley et al., 2005; Chakraborti, 2015; Dreyer and Liebl, 2018). On the other hand, some studies have shown no associations between obesity and the F:B ratio or have shown associations with other taxa, such as the higher relative abundance of taxa like *Actinobacteria* and *Bacteroidetes* (Turnbaugh et al., 2008; Murphy et al., 2010; Clarke et al., 2012; Del Chierico et al., 2018). However, there appears to be an overall agreed upon increase in Firmicutes (Abdallah Ismail et al., 2011) and evidence of gut microbial differences (Turnbaugh et al., 2008; Murphy et al., 2010; Clarke et al., 2012; Del Chierico et al., 2018) in obese individuals, which could be genetically or environmentally induced. There is evidence that gut dysbiosis may induce obesity. In an animal study by Turnbough et al. (Turnbaugh et al., 2006), cecal microbiota from lean and obese mice were transplanted into the gut of germ-free mice. After 2 weeks, the mice that had been transplanted with microbiota from obese mice extracted more calories from their food and had greater fat deposition compared to mice receiving transplants from lean mice (Turnbaugh et al., 2006; Turnbaugh et al., 2008). An earlier study (Shi et al., 2006), also in animals, showed that lipopolysaccharides (LPS), a component of gram-negative bacterial cell walls is produced at higher levels in cases of gut dysbiosis secondary to increased gram-negative bacteria during dysbiosis since gram negative bacteria can tolerate and flourish in an inflammatory environment. Both inflammation and LPS induce a leaky gut which worsens as bacteria translocate across the gaps between the epithelial cells. Increased LPS has been found to be associated with obesity and insulin resistance by binding to CD14 toll-like receptor-4 (TLR-4) and transforming growth factor-β (TGF-β)-mediated pathways (Abreu, 2010; Manco et al., 2010). Earlier animal studies shows that infusion of LPS and associated high levels mimicked the effects of a high fat diet on metabolic and inflammatory parameters (Cani et al., 2007). This finding was later verified in work by Cani et al (Cani et al., 2007), and the term metabolic endotoxemia was coined as the levels of endotoxins observed during obesity are lower than that seen during septic shock. While exact mechanisms linking obesity with these patterns of microbial composition are unknown, the result is increased systemic inflammation, likely secondary to increased gut leakiness and a reduction in microbial metabolites such as short chain fatty acids that have can induce satiety (Utzschneider et al., 2016).

Importantly, studies from humans provide support to the notion that gut dysbiosis may be a driver of obesity. In a small study of 12 obese individuals with lower relative proportions of Bacteroidetes and higher Firmicutes at baseline (as compared to lean controls), the proportion of Bacteroidetes increased over time, mirroring reductions in body weight but not dietary interventions (Ley et al., 2005). Another study, Santacruz et al. (2009) enrolled 36 overweight adolescents in a diet and exercise weight-loss program and found that greater weight loss in response to the lifestyle intervention was associated with the baseline gut microbiome profile prior to the treatment. Specifically, total bacterial count, *B. fragilis*, *Clostridium leptum*, and *Bifidobacterium catenulatum* group counts were significantly higher (P<0.001-0.036) while *C. coccoides*, *Lactobacillus*, *Bifidobacterium breve*, and *Bifidobacterium bifidum* counts were significantly lower (P<0.001-0.008) in the high weight-loss group (> 4kg weight loss) than in the low weight-loss group (<2 kg weight loss) both before and after the lifestyle intervention.

While a specific microbiome signature associated with obesity may not be consistent across different studies, likely due to several factors including dietary habits and environmental factors, it is the metabolic effect exerted through microbial metabolites that plays a larger role. The gut microbiota interacts with the host through several mechanisms including the production of short chain fatty acids (SCFAs), including acetate, butyrate and propionate, that are the byproducts of fermentation of non-digestible starches and fibers in the large intestine. SCFAs serve as energy substrates (Bergman, 1990; Brown et al., 2003; Louis et al., 2010; Blaut, 2015) and mediate crosstalk between bacteria and host metabolism (Manco et al., 2010). For example, they activate the mucosal G protein-coupled receptors (GPR) GPR-43 and GPR-41 to regulate secretion of incretin hormones such as glucagon like peptide-1 (GLP-1) (Nøhr et al., 2013; Kimura et al., 2014; Le Poul et al., 2003) and peptide YY (PYY) (Carvalho and Abdalla Saad, 2013), and adipose tissue-derived leptin. Incretin hormones, including GLP-1 and PYY are normally released in response to a meal and augment the secretion of insulin, thus stimulating a decrease in blood glucose levels. In addition, these hormones can act on the hypothalamus to promote satiety and reduce food intake (Carvalho and Abdalla Saad, 2013). SCFAs have also been shown to have anti-inflammatory actions (Carvalho and Abdalla Saad, 2013), which could be important, as obesity which is characterized by chronic low-grade inflammation.

Other metabolic effects of the gut microbiome on obesity have been described. For example, some studies suggest that a bile acid (BA)-gut microbiome axis contributes to insulin sensitivity and obesity (Sun et al., 2018). Consistent with this idea, the gut microbiome plays a major role in BA metabolism (Hullar et al., 2014; Ridlon et al., 2014) and evidence suggests that BAs are involved in the regulation of glucose homeostasis and insulin sensitivity (Kars et al., 2010; Haeusler et al., 2013; Kuipers et al., 2014; Wewalka et al., 2014). It is thought that BAs can impact insulin sensitivity *via* effects on the nuclear farsenoid X receptor (FXR) and the membrane-bound G-protein coupled receptor, TGR5 (Kuipers et al., 2014). Therefore, the gut microbial composition can alter insulin sensitivity by changing the amount and type of secondary BAs formed, thereby affecting FXR and TGR5 signaling. Indeed, one human study showed that the use of oral vancomycin altered the gut microbiome and caused a decrease in secondary BAs that were associated with worsened insulin sensitivity (Vrieze et al., 2014). Another study showed that oral metformin altered the gut microbiome composition in obese adults with type 2 diabetes (T2D) which was associated with secondary BA and FXR changes, resulting in improved insulin sensitivity (Sun et al., 2018).

### T1D, Obesity, and the Gut Microbiome

Type 1 Diabetes Exchange clinic registry data between the years of 2010 and 2012 indicate that the prevalence of obesity (BMI ≥95th percentile for age and sex) was 13.5% overall in adolescents with established T1D and was higher in black/African American (17.9%) and Hispanic/Latinx (15.9%) children (Minges et al., 2017). Epidemiological studies have also shown increased prevalence of obesity among adults with T1D (Nathan et al., 2009; Conway et al., 2010). Further, some data show an increasing rate of obesity among people with T1D compared to general population trends (DuBose et al., 2015). Obese children progress faster to T1D and display differences in endogenous insulin secretion compared to their lean counterparts (Redondo et al., 2012; Ferrara et al., 2017). Obesity also reduces insulin sensitivity (Redondo et al., 2012; Ferrara et al., 2017), which is associated with higher exogenous insulin needs, chronic inflammation, and higher risk for hypoglycemia, dyslipidemia, and increased risk of long-term complications (Redondo et al., 2016; Corbin et al., 2018).

The causes for obesity in T1D are likely multifactorial and are not completely understood. Potential factors can include the fact that insulin therapy stimulates appetite, genetic predispositions, dietary composition, the frequent need to treat hypoglycemia with high caloric drinks, and possible disordered eating in this population. However, other potentially unknown environmental factors, including the gut microbiome, should be considered.

Independent of obesity, T1D development is influenced by the intestinal microbiome (Zheng et al., 2018). Children with T1D and islet antibody positive relatives who later progress to T1D exhibit gut dysbiosis and lower gram-positive to gram-negative gut bacterial ratios compared to healthy children (Brugman et al., 2006; de Kort et al., 2011; Brown et al., 2011; Giongo et al., 2011; de Goffau et al., 2013; Murri et al., 2013; de Goffau et al., 2014; Mejía-León et al., 2014; Kostic et al., 2015; O'Callaghan and van Sinderen, 2016) (**Table 2**). These imbalances could create a pro-inflammatory milieu and gut leakiness, thereby activating the autoimmune process. Gut microbiome changes including increased virulence factors, elevated phage, prophage, motility genes, and higher amplitude stress responses have been also identified in children who have or are progressing towards T1D (Brugman et al., 2006; de Kort et al., 2011; Brown et al., 2011; Giongo et al., 2011; de Goffau et al., 2013; Murri et al., 2013; de Goffau et al., 2014; Mejía-León et al., 2014; Kostic et al., 2015; O'Callaghan and van Sinderen, 2016).

**Table 2:** Table highlighting some similarities and differences in bacterial species seen in obesity and in type 1 diabetes.

Species	Obesity	T1D
*Firmicutes*	Increased (elevated *Firmicutes : Bacteroidetes)*	Decreased (reduced *Firmicutes : Bacteroidetes)*
*Bacteroidetes*	Decreased (elevated *Firmicutes : Bacteroidetes)*	Increased (reduced *Firmicutes : Bacteroidetes)*
*Actinobacteria*	Increased	unknown
*Bifidobacterium*	Decreased	Decreased
*Clostridium*	Increased	Increased

These microbiome changes, however, appear to be distinct from the changes seen in individuals with obesity. For example, animal and human studies suggest higher relative abundance of taxa (such as *Actinobacteria* and *Bacteroidetes*) associated with and linked to changes in BA and steroid acid biosynthesis in obese adolescents and adults (Del Chierico et al., 2018), while T1D studies show lower counts of *Firmicutes* and *Bifidobacteria* (O'Callaghan and van Sinderen, 2016; Zheng et al., 2018).

Currently, the pathways through which the microbiome influences metabolic health in T1D are not fully understood. However, similar to obesity and other diseases, the gut microbiome may influence T1D, in part, through the production of specific metabolites. Children with T1D have lower circulating SCFAs (Zheng et al., 2018), and the gut microbiome of children with T1D contains fewer bacteria (fermenters) that produce butyrate. In addition to effects mentioned earlier, butyrate has anti-inflammatory actions and has effects that enhance the immune regulatory responses (Brugman et al., 2006; de Kort et al., 2011; Brown et al., 2011; Giongo et al., 2011; de Goffau et al., 2013; Murri et al., 2013; de Goffau et al., 2014; Mejía-León et al., 2014; O'Callaghan and van Sinderen, 2016). Together these data suggest that a potentially common pathway through which the microbiome impacts metabolic health in persons with T1D, non-diabetic individuals, and in obese individuals is through modulating SCFA production.

It appears that gut microbiome changes are associated *either* with T1D development (Brugman et al., 2006; de Kort et al., 2011; Brown et al., 2011; Giongo et al., 2011; de Goffau et al., 2013; Murri et al., 2013; de Goffau et al., 2014; Mejía-León et al., 2014; Kostic et al., 2015; O'Callaghan and van Sinderen, 2016; Zheng et al., 2018) *or* with obesity in non-diabetic cohorts (Cani et al., 2007; Turnbaugh et al., 2009; Musso et al., 2010; Del Chierico et al., 2018; Peters et al., 2018). However, the gut microbiomes of lean and obese individuals with T1D have never been compared. Further, it is not known whether metabolic differences seen in obese individuals with T1D compared to lean individuals with T1D are associate with gut microbiome and metabolite differences. Notably, there is little information concerning SCFA and BA levels in T1D individuals who are obese despite the known mechanistic roles of these molecules in the pathophysiology of other gut microbiome dysbioses and conditions. A study by our group is currently underway aiming to address the lack of knowledge concerning the composition and potential role of the gut microbiome and microbial metabolites in lean and obese T1D individuals and potential associations with metabolic health. Data from this study could yield mechanistic insights that can be translated into novel therapeutic modalities.

### Potential Interventions and Metabolic Pathways to Consider

**Figure 1:**
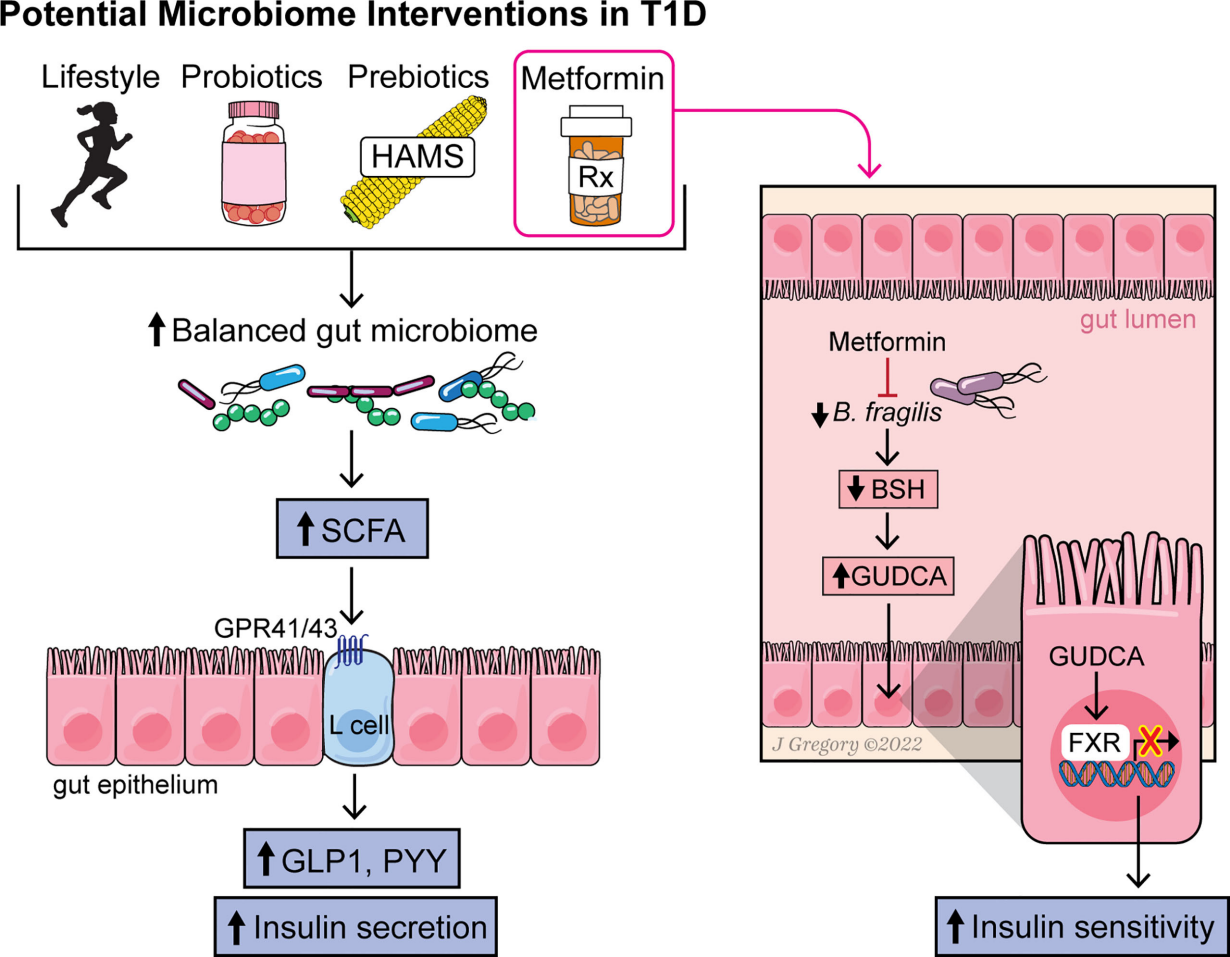
Figure summarizing the effect of various interventions on the gut microbiome, microbial metabolites and pathways leading to increased insulin sensitivity and secretion.

Several interventions to modulate the gut microbiome and disease state have shown promising results in either T1D or obesity and might be worth considering in an obese T1D population. Yet questions remain as to whether modulation of the microbiome could have beneficial effects in a population of individuals with T1D and obesity. Here, we highlight a few potential options (**Figure 1**).

#### Lifestyle Interventions

First, one must consider lifestyle interventions as an easy and safe intervention. Nutrition and exercise are important therapeutic approaches to improve glycemic control and manage body weight. There are consensus guidelines on the management of obesity and T2D (American Diabetes Association, 2020) but these guidelines are lacking when addressing obesity in a T1D population. Restoration of the gut microbiome balance has been shown to be influenced by or associated with a healthy BMI and increased physical activity levels in a population of adults with T1D (Stewart et al., 2017). Therefore, increased physical activity levels and a healthy diet along with the associated improvements in body composition and glycemic control could be an easy target to restore the dysbiosis that is likely present in T1D individuals with obesity. However, as the response to these interventions can vary from one individual to another (Zeevi et al., 2015), an important first step would be to understand the role of the gut microbiome in driving host energy metabolism and macronutrient balance (the balance between dietary energy harvest and expenditure) in this population in order to provide a more personalized lifestyle approach. It is postulated that there is a microbe-mediated increase in energy uptake that has been estimated to account for 10% of the energy intake among those consuming a western diet (McNeil, 1984).

#### Probiotics

Probiotics are viable microorganisms that have health promoting effects on the host when administered in adequate amounts as food ingredients (Rastall et al., 2005; Shen et al., 2013; Davis, 2016). Studies have shown an effect for probiotics containing Lactobacillus, Bifidobacterium, or Saccharomyces on weight loss and/or fat deposition in overweight adults (Rastall et al., 2005; Crovesy et al., 2017). In addition, multi-species probiotics restore gut microbiota profiles and promote epithelial tight junction integrity and reduce inflammation (Carvalho and Abdalla Saad, 2013; Chibbar et al., 2017), thereby preventing fat accumulation and weight gain (Everard et al., 2013; Davis, 2016). Multi-strain probiotics are available as a commercial probiotic mixture of *lactobacilli* (*L. casei, L. plantarum, L. acidophilus, and L. delbrueckii subsp. bulgaricus*); *bifidobacteria (B. longum, B. breve, and B. infantis*); and *Streptococcus* (*S. salivarius subsp. thermophilus*) (Shen et al., 2013; Chibbar et al., 2017). Multi-strain probiotics have been also studied in T1D. An Italian study showed that supplementation with *Lactobacillaceae*-enriched multi-strain probiotics, given alone or along with retinoic acid, decreased the risk of developing diabetes in non-obese diabetic (NOD) mice (Dolpady et al., 2016). In humans, multi-strain probiotics have also been studied in the modulation of the immune system in 25 unaffected siblings of T1D patients who are considered at risk for T1D development. Results show that the probiotic was safe and well tolerated and that systemic inflammation was modestly reduced in response to the probiotic intake (Cabrera et al., 2022). In this study, markers of inflammation were significantly reduced after 6-weeks of probiotic supplementation (p = 0.017). For example, probiotic-associated decrease in the ratio of memory: naïve CD4+ cells were demonstrated, consistent with lowering of systemic inflammation. They also noted a post-supplement enrichment of the family *Lachnospiraceae*, producers of the anti-inflammatory butyrate. Therefore, this multi-strain probiotic ‘mixture’ of *lactobacilli* might also have potential use in an obese T1D population.

#### Prebiotics

Prebiotics are non-digestible food ingredients capable of selectively stimulating growth and/or activity of fermenters and high SCFA producers, thereby providing health-promoting effects on host energy balance (Lim et al., 2005; Roberfroid et al., 2010). Prebiotics can, therefore, modify the gut microbiota to mitigate the risk of dysbiosis and associated gut and systemic pathologies.

One such prebiotic that could be promising in an obese T1D population is high amylose maize starch (HAMS), which has been shown to be effective in T1D as well as in overweight and obese adults, making it attractive in an obese T1D population (Maki et al., 2012; Dainty et al., 2016; Mariño et al., 2017; Stewart and Zimmer, 2018). HAMS is a versatile and well-tolerated source of indigestible dietary fiber with selective fermentation properties that shift the gut microbiome profile towards fermenters and SCFA production. In non-diabetic adults, HAMS consumption showed lower post-prandial glucose levels, along with improved insulin sensitivity and secretion. In a human study assessing the effects of 2 levels of intake of HAMS [15 or 30 g/d (double-blind)], as compared to control starch intake (0 HAMS) for 4-wk periods separated by 3-wk washouts, consumption of 15–30 g/d of HAMS improved insulin sensitivity significantly in men (Maki et al., 2012). Another human randomized, double-blind, controlled study (Stewart and Zimmer, 2018) in 28 non-diabetic healthy adults who consumed either a high fiber scone containing a novel chemically modified high amylose maize starch or a low fiber control scone without the maize starch, the consumption of the high fiber scone significantly reduced postprandial glucose and insulin incremental areas under the curves (43–45% reduction, 35–40% reduction, respectively), postprandial glucose, and insulin maximum concentrations (8–10% and 22% reduction, respectively), suggesting a beneficial glycemic effect along with improved insulin sensitivity. Lastly, in a study of 24 men and women at risk for T2D, consumption of HAMS resulted in a significantly lower fasting, 2-h, and 3-h insulin incremental areas under the curve during an oral glucose tolerance test, while fasting insulin resistance measures using the homeostasis model assessment of insulin resistance were significantly lower (Dainty et al., 2016). However, gut microbiome changes were not assessed in any of the above studies.

Marino et al. (Mariño et al., 2017) found that after feeding non-obese diabetic mice (mouse models of T1D) diets rich in high-amylose maize starch designed to release large amounts of the SCFAs acetate and butyrate after colonic bacterial fermentation, key features of T1D (such as disease progression, markers of inflammation and islet autoimmunity) were lower and were associated with a shift in the gut microbiome profile towards fermenters with higher blood and fecal concentrations of the microbial metabolites acetate and butyrate. The mice were also highly protected from diabetes, even when the fiber was administered following the development of islet-specific immune autoreactivity. The non-obese diabetic mouse (NOD) disease process, although similar in some aspects to human T1D, it is somewhat different in that the incidence for females is much higher than for males and the appearance of insulitis in mice and some patients is also different (Chen et al., 2018). Given this, the same group later showed that 6 weeks of the prebiotic supplementation in adults with longstanding T1D was associated with increased SCFAs in stools and plasma, and that subjects with the highest SCFA concentrations exhibited the best glycemic control. They also showed that circulating B and T cells developed a more regulatory phenotype following the supplementation (Bell et al., 2022).

Another prebiotic of interest is inulin. Inulin is a prebiotic that has been studied in both T1D and obesity separately and that targets gut microbiota and thereby influences microbial composition and activity. In a double-blind, randomized, placebo-controlled crossover study of 14 healthy, overweight to obese men, inulin promoted SCFA production with significantly higher plasma acetate after ingestion compared to placebo (van der Beek et al., 2018). A randomized, placebo-controlled trial in 38 children aged 8 to 17 years old with T1D using either placebo or a prebiotic oligofructose-enriched inulin for 12 weeks showed that C-peptide was significantly higher (P=0.029) in the prebiotic group, along with a modest improvement in intestinal permeability (P=0.076) (Ho et al., 2019). Although the authors did not look at microbial metabolites and metabolic pathways, they reported a significant increase in the relative abundance of Bifidobacterium (a known bacterial fermenter) in the prebiotic group.

#### Pharmacological Interventions

The effect of metformin on the microbiome has also been studied. Metformin is a first-line drug used to treat T2D, as it improves insulin sensitivity and inhibits hepatic gluconeogenesis. In adolescents with T1D, metformin use, in addition to insulin therapy, reduced total daily insulin doses (Libman et al., 2015). In another study, metformin improved whole-body and peripheral insulin sensitivity in T1D adolescents who were overweight and obese (Cree-Green et al., 2019). Results of preclinical animal studies suggest that metformin changes gut microbiome composition (Lee and Ko, 2014; Zhang et al., 2015). More recently, it has been shown that the therapeutic benefit of metformin may be due, in part, to alterations in the gut microbiome that modulate host energy metabolism (Ridlon et al., 2014; Wu et al., 2017; Lee et al., 2021). Several studies of different ethnic populations with T2D and healthy individuals have demonstrated that metformin alters gut microbiome composition and is associated with changes in SCFA, BA, and C-peptide levels (Lee et al., 2021). A study in humans by Sun et al. (Ridlon et al., 2014) showed that exposure to metformin for 3 days in adults with newly diagnosed T2D (who were treatment naïve) markedly reduced *Baceteroides* abundance in fecal samples, with the most striking decrease seen in *Bacteroides (B.) fragilis*. Metformin treatment also increased levels of the secondary BA glycoursodeoxycholic acid (GUDCA) in the gut through decreasing the abundance of *B. fragilis* and its bile salt hydrolase (BSH) activity in the intestines (Ridlon et al., 2014). In turn, GUDCA functioned as an FXR antagonist in the gut to improve insulin sensitivity and glycemic control (Ridlon et al., 2014). Elevated tauroursodeoxycholic acid (TUDCA) levels were also observed. These findings suggest a key role for microbiota-derived changes in GUDCA and the FXR axis as a mediator of metformin’s actions.

Work from other groups (Lee et al., 2021) support key findings from the study by Sun et al. For example, in one double-blind study (Wu et al., 2017), individuals with treatment naive T2D were randomized to placebo or metformin for 4 months, and then their gut microbiomes were analyzed. The relative abundance of more than 80 bacterial strains showed alterations in the metformin arm after 2 and 4 months compared to a change in only one strain in the placebo arm. These results were further verified in a subset of the placebo group that switched to metformin 6 months after the trial started. Lastly, the authors transferred fecal samples obtained before and 4 months after treatment from metformin-treated donors to germ-free mice. This transfer resulted in improved glucose tolerance in the mice that received the metformin-altered microbiota.

Metformin also promotes colonization by SCFA-producing bacteria such as *Akkermansia* (Utzschneider et al., 2016; de la Cuesta-Zuluaga et al., 2017). SCFAs in turn can regulate the secretion of GLP-1, which has been shown to enhance the metabolic effects of BAs that signal through another receptor (the Takeda G-protein coupled receptor 5, or TGR5) (Thomas et al., 2009), indicating synergy between metformin’s effects on SCFAs and its effects on BAs. These therapeutically relevant insights into the emerging field of altered gut microbiota-mediated BA and SCFA signaling and T2D pathophysiology provide the rationale for considering metformin use to modify the gut microbiome in T1D individuals with obesity.

#### Fecal Microbiome Transplantation

Fecal microbial transplantation (FMT) is a procedure in which fecal matter is collected from a healthy donor and placed into the gastrointestinal tract of a patient. Recent studies have explored the use of FMT in T1D as well as in obesity. For example, in a double-blind randomized placebo control pilot study (Allegretti et al., 2021) using FMT in 22 obese adults, significant improvements were seen in glucose area under the curve (AUC) at 12 weeks compared to baseline, and in the insulin AUC at 6 weeks compared to baseline in the FMT group compared to placebo. These findings suggest that FMTs may have a potential role in preventing the development of metabolic syndrome in patients with obesity. However, the same group (Allegretti et al., 2020) had shown earlier that although FMTs were safe and tolerable in this group of participants, it did not reduce BMI. They did show, however, that FMTs led to sustained changes in the intestinal microbiome and BA profiles that were similar to those of the lean donor.

A recent study (de Groot et al., 2021) of 20 new onset T1D patients ages 18-30 years and within 6 weeks of diagnosis, showed that stimulated C-peptide levels (as measured by mixed meal tolerance testing) were significantly preserved in the autologous FMT group compared with the healthy donor FMT group at 12 months. These authors also found that several microbial metabolites and bacterial strains were linked to this preserved residual β-cell function. Specifically, they found that the change in the relative abundance of *Desulfovibrio piger* correlated positively with change in fasting C peptide (p=0.009) and the plasma metabolite*1-myristoyl-2-arachidonoyl-GPC* correlated significantly with changes in fasting C peptide (p=0.012). It is rather interesting that the autologous FMT group had a higher preserved stimulated C-peptide, though the authors explain this is not surprising given that FMTs can affect homeostasis by introducing fecal microecology into the much less densely populated small intestine since FMTs in this study were introduced *via* a nasoduodenal tube. It is, in the opinion of the authors of this review, that autologous FMTs were likely associated with better recipient engraftment.

However, the FDA recently issued a warning against FMTs (US Food and Drug Administration, 2022), commonly used to treat chronic *Clostridium Difficile* infections, given that two immunocompromised individuals developed severe life-threatening infections from an antibiotic resistant strain of *E. coli* present in the donor stool, resulting in one of the two dying. FMT donors are screened for chronic and serious transmissible infections, such as HIV and hepatitis, but their samples are not tested for drug resistant strains. Yet, with more awareness and understanding of this procedure and its safety, FMTs may hold some promise in treatment of obese T1D individuals due to a potentially longer sustained effect on recipient colonic colonization (or decolonization of pathogenic strains) compared to the use of probiotics, for example, that tend to produce a more short-term change in the gut microbiome composition.

## Conclusions

Obesity is a concerning and increasing health problem among individuals with T1D, and it is important to consider the potential role of the gut microbiome in obesity in these individuals (Musso et al., 2010). As the obesity epidemic can be viewed as an extension of the hygiene hypothesis, which stipulates that improved sanitation, widespread antibiotic use, and dietary habits in developed countries may predispose to metabolic diseases, the deviant gut microbiota may mediate these associations. In this case, this population of individuals with T1D and obesity should be viewed as facing a ‘double whammy’ given the hygiene hypothesis has been speculated to contribute to autoimmune disease development, hence targeting the gut microbiome is even more compelling. Several questions remain unanswered. Does the gut microbiome of obese individuals with T1D mimic that of obese non-diabetic individuals or that of T1D individuals or a combination of both? Are the microbiome-metabolite-metabolic pathways similarly involved in obese T1D individuals? Can we ascertain causality between the gut microbiome and obesity in T1D? And finally, do lifestyle changes, certain supplements and medications, or FMTs produce similar effects in this population and who would be a good candidate for each intervention?

## Future Directions

Importantly, future studies should examine the gut microbiome of individuals with T1D and obesity and ascertain the nature of the relationship, focusing on the gut microbial functional capacity in this population to better understand the hormonal, immunomodulatory, and metabolic mechanisms underlying the microbiome-host interactions. This would allow for a more targeted intervention aimed at deficient metabolic pathways and restoration of the functional capacity of the disrupted microbiome. It will be essential to design future clinical trials to assess the health benefits derived from microbiome modulating interventions on the treatment of obesity in individuals with T1D and assess the long-term safety of gut microbiota manipulation.

## Author Contributions

HI and CE-M each contributed to the conceptualization of the review, writing and editing. All authors contributed to the article and approved the submitted version.

## Funding

Research in Dr. Evans-Molina’s lab is supported by National Institute of Diabetes and Digestive and Kidney Diseases grants R01DK093954, R01DK127236, U01DK127786, R01DK127308, UC4DK104166 (to C.E.-M.), U.S. Department of Veterans Affairs Merit Award I01BX001733 (to C.E.-M.), and gifts from the Sigma Beta Sorority, the Ball Brothers. Dr. Ismail's work was supported by Grant 2021258 from the Doris Duke Charitable Foundation through the COVID-19 Fund to Retain Clinical Scientists collaborative grant program and was made possible through the support of Grant 62288 from the John Templeton Foundation. This publication was also supported by a Pilot and Feasibility Award within the CDMD NIH/NIDDK Grant Number P30 DK097512.

## Author Disclaimer

The opinions expressed in this publication are those of the author(s) and do not necessarily reflect the view of the John Templeton Foundation.

## Conflict of Interest

The authors declare that the manuscript was written in the absence of any commercial or financial relationships that could be construed as a potential conflict of interest.

## Publisher’s Note

All claims expressed in this article are solely those of the authors and do not necessarily represent those of their affiliated organizations, or those of the publisher, the editors and the reviewers. Any product that may be evaluated in this article, or claim that may be made by its manufacturer, is not guaranteed or endorsed by the publisher.
